# Editorial of Special Issue “Human Pathogenic Fungi: Host–Pathogen Interactions and Virulence”

**DOI:** 10.3390/microorganisms11040963

**Published:** 2023-04-07

**Authors:** Samir Jawhara

**Affiliations:** 1 UMR 8576—UGSF—Unité de Glycobiologie Structurale et Fonctionnelle, Centre National de la Recherche Scientifique, F-59000 Lille, France; samir.jawhara@univ-lille.fr; 2 Institut National de la Santé et de la Recherche Médicale U1285, University of Lille, F-59000 Lille, France; 3 Medicine Faculty, University of Lille, F-59000 Lille, France

Most individuals harbour several species of yeast of the genus Candida, which are considered true symbionts of the human gut microbiota [1,2,3,4]. However, *Candida albicans* is the major fungal species in the human gut [2,3]. *C. albicans* is an opportunistic pathogen responsible for the majority of mucosal and systemic fungal infections. Over recent years, researchers have become increasingly interested in the role of *C. albicans* in modulating colonic inflammation, for two reasons [2]. The first concerns alterations in the gut microbiota, the rupture of epithelial barriers or dysfunction of the immune system, which all favour the transition of *C. albicans* from a commensal to a pathogen [4]. The second concerns its excessive abundance in the gut of patients with Crohn’s disease (CD) [5,6]. CD is a chronic form of inflammatory bowel disease (IBD) that has a multifactorial aetiology, resulting from the interaction of genetic, environmental and microbial factors [7]. Its incidence has been increasing rapidly worldwide, especially in newly industrialised countries, suggesting the involvement of the Western diet in the onset and progression of IBD [8]. Rapid urbanisation in the developing world has been observed to reduce the biodiversity of the gut microbiota [9]. Several studies have shown that gut microbiota dysbiosis is a key factor in the pathogenesis of CD and can be used as a biomarker to distinguish between CD and non-CD [7,10]. A decrease in the abundance and biodiversity of the intestinal microbiota has been observed in CD patients [11]. Different clinical studies have reported that the diversity and richness of fungal populations were significantly higher in the inflamed mucosa compared to the non-inflamed mucosa in CD patients [11]. In a murine model of dextran sulphate sodium (DSS)-induced colitis, *C. albicans* overgrowth was shown to be involved in mucosal damage of the murine gut [6,12]. Additionally, *C. albicans* overgrowth aggravates the inflammatory mediator response in mice with DSS-induced colitis and, reciprocally, these inflammatory parameters increase *C. albicans* overgrowth. A review [2] entitled “How gut bacterial dysbiosis can promote *Candida albicans* overgrowth during colonic inflammation” highlights the role of probiotics, such as *Saccharomyces boulardii* or *Saccharomyces cerevisiae*, in the elimination of *C. albicans* from the gut [13]. *S. boulardii* is a non-pathogenic yeast used as a probiotic strain in the prevention or treatment of intestinal diseases, mainly diarrhoea [14,15]. Clinical and experimental evidence shows that *S. boulardii* may have therapeutic potential in IBD patients [13,16,17]. The effect of fungal fractions, such as β-glucan or chitin, was also addressed in this review, illustrating how these fungal glycan fractions can modulate the immune response and attenuate the inflammatory response in the host [18,19,20]. Treatment of mice with fungal β-glucan or chitin fractions reduced DSS-induced colonic injury and inflammatory mediators in mice, indicating that these soluble glycan fractions boost the immune response against the overgrowth of opportunistic pathogens and help in maintaining intestinal homeostasis [21]. In addition, these fungal glycans also had a beneficial impact on the restoration of the gut microbiota.

IBD patients are highly vulnerable to colonisation/infection with *C. albicans*, complicating IBD treatment. Currently, most anti-inflammatory drugs prescribed do not have antifungal activity. With the objective of identifying new compounds with both antifungal and anti-inflammatory properties, Dumortier et al. explored the effect of N-[2-(p-bromocinnamylamino)ethyl]-5-isoquinolinesulfonamide (H89), a kinase A inhibitor protein on inflammatory cells and on the viability and growth of *C. albicans*, gut inflammation and modulation of the gut microbiota [22]. This study demonstrated that H89 attenuated the migration of macrophages to the site of inflammation and revealed its antifungal effect against *C. albicans* growth [22]. In the DSS-induced colitis model, treatment of mice with H89 reduced intestinal inflammation as well as the expression of inflammatory mediators. H89 also restored the population of anaerobic bacteria, such *as Lactobacillus johnsonii* and *Bacteroides thetaiotaomicron*, and reduced the populations of *Escherichia coli* and *Enterococcus faecalis*, suggesting that the restoration of these anaerobic bacteria is crucial for intestinal homeostasis. Furthermore, H89 was not only able to attenuate the inflammation parameters but also promoted *C. albicans* elimination from the mouse gut, indicating that H89 exhibits dual properties against *C. albicans* growth and inflammation [22].

In line with this study, two anaerobic bacteria, *B. thetaiotaomicron* and *L. johnsonii*, have been observed to decrease significantly in the gut during colonic injury and *C. albicans* colonisation [18]. The restoration of these two anaerobic bacteria promoted the elimination of *C. albicans* from the gut and alleviated the development of colitis in mice [23]. These observations led our team to conduct an experimental study on *B. thetaiotaomicron* and *L. johnsonii*, showing that two fatty acids (oleic acid and palmitic acid) are identified from the interaction between these two anaerobic bacteria and *C. albicans* in contact with intestinal epithelial cells [24]. The treatment of mice with oleic acid and palmitic acid combined together exhibited an anti-inflammatory and antifungal effect in the DSS-induced colitis model [24].

In addition to the gut microbiota, the lung microbiome is critical in maintaining immune homeostasis in the airway mucosa [25]. The alteration of the gut-lung cross-talk is related to high susceptibility to airway pathogenesis and infections. The importance of the gut–lung axis is illustrated in patients with IBD who have a higher prevalence of pulmonary diseases [26,27]. Patients with chronic respiratory disorders (CRDs), such as asthma, chronic obstructive pulmonary disease and cystic fibrosis, display not only a dysbiotic airway microbiota but also the components of gastrointestinal perturbation [28]. Altered immune defence mechanisms, the use of immunosuppressants and the frequent use of antibiotics predispose patients with CRD to fungal colonisation and overgrowth in their lower airways. Caballero et al. highlighted the presence of a lung mycobiome in the lower airways of healthy individuals and patients with CRDs. The lung mycobiome is dominated by different filamentous fungi, including *Aspergillus fumigatus*, and by yeast forms, in particular *C. albicans* [29]. Many of these filamentous fungi are likely transient species that are inhaled from environmental air [29].

It is well known that *C. albicans* is the most frequently encountered Candida species, but the incidence of non-*albicans* species, such as *C. glabrata*, *C. parapsilosis* and *C. tropicalis* has increased over recent decades due to the prolonged use and limited options of antifungal drugs [30]. Recently, *C. auris* has emerged worldwide as a fungal pathogen of increasing concern due to its multidrug-resistance and high mortality rates [31]. Billamboz et al. described that *C. auris* expresses several virulence factors that contribute to pathogenesis, including the transition between blastoconidia and filamentous forms, hydrolytic enzyme production, thermotolerance, biofilm/adhesion to host cells, osmotolerance, filamentation and phenotypic switching [32]. This review also focuses on the different new strategies and results obtained during the past decade in the field of antifungal design against this emerging species, based on a medicinal chemist point of view [32].

Overall, the different observations and data presented and discussed in this Special Issue highlight the involvement of *C. albicans* in the pathogenesis of IBDs. Different preventive or therapeutic strategies, including probiotics (*S. boulardii*), fungal glycan fractions (β-glucans and chitin) and new antifungal and anti-inflammatory drugs against Candida and the inflammatory process are discussed in this Special Issue. Finally, it is well known that Candida is able to adapt to the host environment, benefit from gut dysbiosis and subsequently modulate the immune response. This dysbiosis involves the loss of beneficial microorganisms and an expansion of pathogenic fungi that trigger the pro-inflammatory mediators of the immune response in IBD patients. Regarding this dysbiosis, it is critical to determine which specific bacterial populations are involved in the restoration of intestinal homeostasis, as this would represent a step towards a treat-to-target strategy in IBD patients.
